# Zero-tolerance biosecurity protects high-conservation-value island nature reserve

**DOI:** 10.1038/s41598-017-00450-y

**Published:** 2017-04-10

**Authors:** John K. Scott, Simon J. McKirdy, Johann van der Merwe, Roy Green, Andrew A. Burbidge, Greg Pickles, Darryl C. Hardie, Keith Morris, Peter G. Kendrick, Melissa L. Thomas, Kristin L. Horton, Simon M. O’Connor, Justin Downs, Richard Stoklosa, Russell Lagdon, Barbara Marks, Malcolm Nairn, Kerrie Mengersen

**Affiliations:** 1 Quarantine Expert Panel, 905 St Georges Terrace, Perth, WA 6000 Australia; 2 School of Biological Sciences, The University of Western Australia, Crawley, WA 6009 Australia; 3 CSIRO Land and Water, Private Bag 5, P.O Wembley, WA 6913 Australia; 4 Chevron Australia Pty Ltd, 250 St Georges Terrace, Perth, WA 6000 Australia; 5 Department of Agriculture and Food Western Australia, 3 Baron-Hay Court, South Perth, WA 6151 Australia; 6 Department of Parks and Wildlife Western Australia, 17 Dick Perry Avenue, Technology Park, Western Precinct, Kensington, WA Australia; 7 87 Rosedale St, Floreat, WA 6014 Australia; 8 Veterinary and Life Sciences, Murdoch University, Murdoch, WA 6150 Australia; 9 4 Devon Rd, Swanbourne, WA 6010 Australia; 10 E-Systems, 205 Davey Street, Hobart, TAS 7000 Australia; 11 Australian Research Council Centre of Excellence in Mathematical and Statistical Frontiers, School of Mathematical Sciences, Queensland University of Technology, 2 George St, Brisbane QLD, 4001 Australia

## Abstract

Barrow Island, north-west coast of Australia, is one of the world’s significant conservation areas, harboring marsupials that have become extinct or threatened on mainland Australia as well as a rich diversity of plants and animals, some endemic. Access to construct a Liquefied Natural Gas (LNG) plant, Australia’s largest infrastructure development, on the island was conditional on no non-indigenous species (NIS) becoming established. We developed a comprehensive biosecurity system to protect the island’s biodiversity. From 2009 to 2015 more than 0.5 million passengers and 12.2 million tonnes of freight were transported to the island under the biosecurity system, requiring 1.5 million hrs of inspections. No establishments of NIS were detected. We made four observations that will assist development of biosecurity systems. Firstly, the frequency of detections of organisms corresponded best to a mixture log-normal distribution including the high number of zero inspections and extreme values involving rare incursions. Secondly, comprehensive knowledge of the island’s biota allowed estimation of false positive detections (62% native species). Thirdly, detections at the border did not predict incursions on the island. Fourthly, the workforce detected more than half post-border incursions (59%). Similar approaches can and should be implemented for all areas of significant conservation value.

## Introduction

Biological invasions are a major threat to conservation areas, but the effectiveness of biosecurity systems is notoriously difficult to predict due to the lack of data on entry and establishment of organisms^1–3^. While failed invasions of organisms crossing borders are rarely considered in biosecurity management and ecological research^4^, they are critical to understanding invasion success. The success rate of biological invasions is extremely difficult to measure because the number of unsuccessful invasions is generally unknown^2^. While some governments collect information on species detected at border controls, these data are rarely made available and they do not inspect 100% of all materials arriving^5^. Recommendation 1 of the National Research Council of the United States^5^ called for detailed recording and analysis of border detections; but no studies to date anywhere in the world have adequately addressed this recommendation. Here we show the effort required to establish what we believe to be one of the world’s most effective and comprehensive biosecurity systems, with specific focus on the pathways involved in invasive species’ introductions and the importance of workforce participation. This project included inspection and verification for all cargo items arriving at the border, which enabled us to provide a comprehensive analysis of the numbers and types of organisms crossing a biosecurity barrier. The objective of the biosecurity system (the Quarantine Management System – QMS) is to protect Barrow Island (BWI), an exceptionally high-conservation-value nature reserve.

### Gorgon Project

The Gorgon Project (construction and operation of a liquefied natural gas plant) is the largest single resource development in Australia^6^ and one of the largest in the world. Under Western Australian legislation BWI (235 km^2^), located off the north-west coast of Australia, is a Class A Nature Reserve, the highest level of protection available under state law. The island’s biodiversity conservation values include mammal species that are extinct or threatened on the mainland^7^, and threatened bird, reptile and troglofauna species, as well as numerous endemics. Since 1964 oilfield operations on BWI have restricted access and implemented basic biosecurity procedures^8^. The island has retained its high conservation values with few non-indigenous species (NIS) present^7, 8^. At the commencement of the Gorgon Project in 2009, BWI was one of the largest land masses in the world with no established non-indigenous vertebrates. In contrast, nearby islands have up to a third of their area dominated by non-native flora^9^, carried introduced rodents^10^ or have lost native mammals and birds due to invasive predators^11^. Black rats and domestic mice had been introduced to BWI but were eradicated^12^ before the Gorgon Project commenced. A government condition required that the Project prevent the introduction of any NIS to BWI and surrounding waters^13^. This requirement, and a lack of data on the base-rate of invasions or which organisms posed a biosecurity threat^2, 14–16^ mandated that the Project implement an exceptionally high level of biosecurity. The QMS required approval by government on the advice of a Quarantine Expert Panel, which included independent experts, and which continues to monitor its implementation^13^.

During the >45-year period prior to the Gorgon Project, introductions and establishments included 32 invertebrate and 16 plant species^17^. Over this time the average annual freight to the BWI oilfield was 2,300 tonnes. During the six years from September 2009 to September 2015 total freight to BWI from 20 countries world-wide was 12,162,038 tonnes (Table S1). There were 12,332 flights transporting 693,781 passengers from Perth or Karratha, with over 53,000 workers trained on biosecurity requirements. This six year period covers the main construction phase of the Gorgon Project.

## Results

Without exception, 100% of cargo was subjected to pre-border cleaning, treatment, packaging and inspection prior to moving to the island, and verification upon arrival at the BWI border. A total of 1,472,379 hours of biosecurity inspections was implemented over six years representing more than 600,000 separate inspections after cleaning and treatment of cargo (Table S1). The intensity of inspection effort is larger by orders of magnitude than any previously reported^18^. For example, in USA it is estimated that 76.5% of incoming shipments of plants infested with pathogens pass the ports undetected^19^. Around 10% of aircraft contained live insects in the cargo and interior during a sampling over one year (1998–1999) of a random sample of 1% (730, 2 per day) of aircraft arriving at Miami International Airport, Florida^20^. Despite this, the records maintained by USDA APHIS since 1985 contains more than 500,000 insect interception records^21, 22^. About 72% of infested live plants imported in 2009 passed through the USA border undetected^23^. In contrast to the 100% inspection regime, it is estimated that only 2% of international cargo is inspected on entry into USA^5^, with inspections largely targeted at high risk material.

### Types and numbers of detections

**Figure 1:**
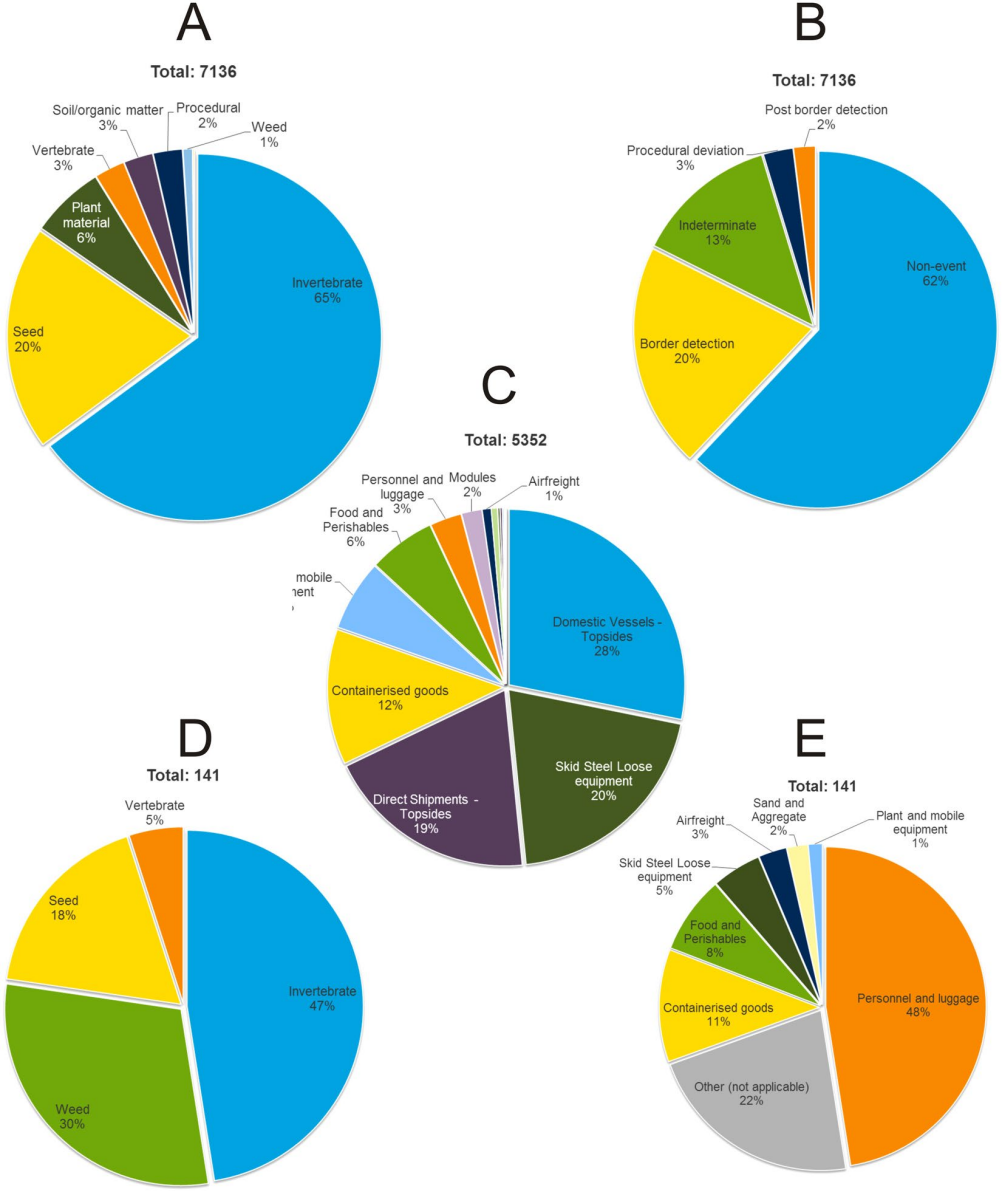
Percentage of border and post-border detections from September 2009 to September 2015 in different categories. (**A**) type of organism (e.g. seed) detected; (**B**) classification of detections; (**C**) detections attributed to pathways (detections that could not be attributed to a pathway were removed); (**D**) post-border detections attributed to pathways; and (**E**) organisms identified in post-border detections. Pie chart segments <5% not labelled. For definitions of biosecurity system categories see main text and Supplementary Methods. Indeterminate refers to those specimens that could not be classified as either indigenous or non-indigenous.

Border and post-border inspections detected organisms 7,136 times, mostly invertebrates and seeds (Fig. 1A). A detection was either specimens of an organism, multiple specimens of an organism or a combination of species found together. However, almost two-thirds of the detections were false positives, the organisms were either dead or unviable, or were native to BWI (Fig. 1B, Table S3). Two factors caused false positives in border and post-border sampling: BWI indigenous species contaminating cargo prior to final inspection, and natural immigration (dispersal to the island by wind or sea, estimated at 1.5% of detections). The flora and fauna of BWI are exceptionally well known as a result of baseline studies undertaken by the Project and previous studies^24, 25^. This provided us with a quantification of the relative frequency of false positive records within a biosecurity management system.

### Pathways for introduction and detection frequency

**Figure 2:**
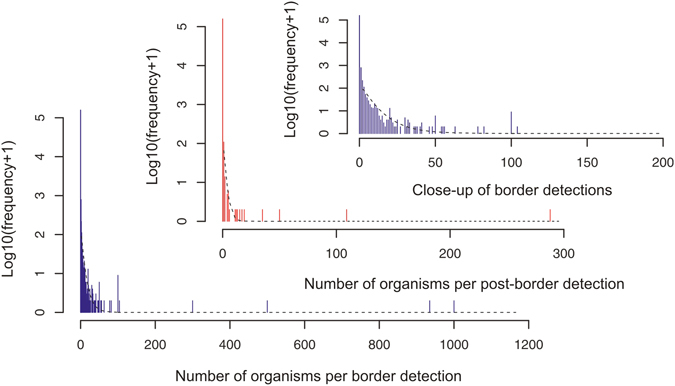
Frequency plot of the number of individual Non Indigenous Species found in 134,265 inspections with 1411 border detections (blue) and 141 post-border detections (red) between 2009 and 2015. Black line indicates fitted mixture distributions comprising a log-normal (LN) density for detections of less than an extreme number of organisms, and point probability masses for detections of zero organisms (I_*i*=*0*_), single organisms (I_*i*=*0*_) (for post-border surveillance) and extreme numbers of organisms (thresholds *l* of 100 and 50 for border and post-border surveillance, respectively). This last mixture component (for extreme numbers of organisms) is depicted as a uniform U(*l,u)* (*0* < *l* < *u)* probability bounded by the threshold *l* to the modelled potential maximum number of organisms *u*, estimated as *u* = ((*k* + *1*)/*k*)(*m* − *1*), where *k* is the sample size and *m* is the sample maximum. Note that these estimates are indicative only, given sensitivity to the choice of threshold and the assumption of uniform, independent large detections. The third inset is a close-up of the fitted mixture distribution for the post-border detections of less extreme numbers of organisms. The corresponding model is given by ${y}_{i}={w}_{1}{I}_{i=0}+{w}_{1}{I}_{i=1}+{w}_{3}\,\mathrm{LN}(\mu ,{\sigma }^{2})+{w}_{4}U(l,u)$ where *w*_*j*_ denote the weights for the four components of the mixture, $j=1,\mathrm{..},4,{w}_{j} > 0,\,\sum {w}_{j}=1$. Note that for a three-component mixture, w_3_ = 0.

All detections were attributed to pathways (Fig. 1C). About half were associated with importation of large structural materials, as the large surfaces involved naturally collected organisms present in the air whilst in transit to BWI. At the border, most inspections recorded zero organisms and most detections of NIS were of single organisms (Fig. 2). On average a detection occurred every 1043 hours of inspection effort at the border.

For the border detections, the standard Poisson and zero-truncated Poisson distributions provided a very poor fit to the data (Fig. 2, Table 1) compared with the negative binomial and the log-normal distributions (Table 1). For the border detections, including null inspections, the negative binomial model produced a better fit than the standard Poisson model (Table 1). Adding a zero-inflation term to the negative binomial distribution improved the fit substantially (evidence <0.5), but three-component mixture distributions provided further improvement (19 extreme values with 100+ organisms; AIC_MP_ = 12,190; AIC_NB_ = 6,759, Table 1). Indeed, by accounting for excess zeros and extreme values in this way, the mixture log-normal distribution provided an even better fit (AIC_MLN_ = 5,529, Table 1). A further component to allow for the many detections of single organisms was also identified, although the AIC value (3,143) is not able to be compared with those cited above.

**Table 1:** Models of the data in Fig. 2 and values of AIC.

Theoretical model	AIC border	AIC post-border
Including zero detections		
Poisson	135,374	9,820
Negative Binomial	27,276	1,991
Zero-inflated Poisson	56,756	5,219
Zero-inflated Negative Binomial	24,460	2,318
Excluding zero detections		
Standard Poisson	38,215	3,406
Standard Negative Binomial	7,824	733
Zero-truncated Poisson	38,208	3,406
Zero-truncated Negative Binomial	6,605	1,324
Log-Normal	5,977	510
Excluding zero detections and extreme values		
Mixture Poisson	12,190	957
Mixture Negative Binomial	6,759	590
Mixture Log-Normal	5,529	424

### Organisms detected

**Figure 3:**
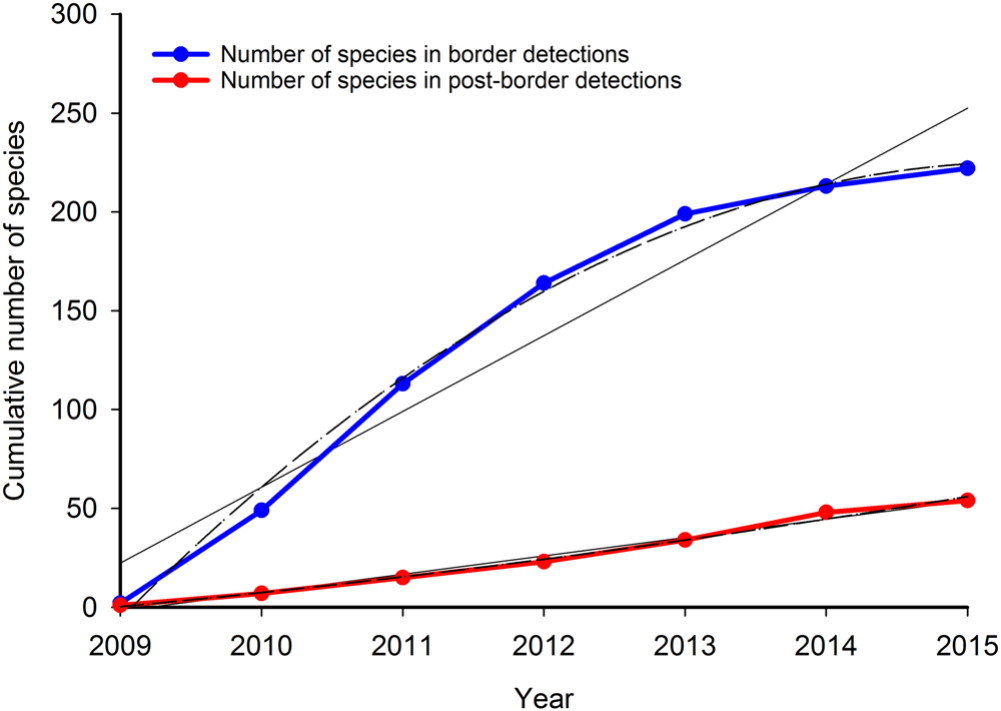
Cumulative number of Non Indigenous Species detected over the five years of QMS operation in border and post-border detections. The observed cumulative number of species in border detections (blue line) and post-border detections (red line), with linear (solid black), quadratic (dashed black) and cubic (dotted black) generalised linear models fitted to the number of counts per year, assuming Poisson distributed residuals. Nonlinear models were preferred in both cases, providing statistical support for a downward trend in number of species detected in more recent years. (Cubic model preferred for border detections: AIC value for linear model 157.4, quadratic 65.6, cubic 49.9; all p-values < 1.0E-3 for all models. Quadratic model preferred for post-border detections: AIC value for linear model 40.1, quadratic 32.2, cubic 37.2; all p-values < 0.03 for linear and quadratic models; all p-values > 0.05 for cubic model).

A wide range of organisms detected (plants, insects, snails, geckoes) supported the initial risk management strategy^26^, which made no assumptions on the types of organisms that would cross the biosecurity border. The five most frequently detected NIS were wind dispersed plant seeds (Table S4). After two years most of the commonly detected NIS had been recorded (Table S4), although the total number of NIS (>200 species) continued to increase reflecting high diversity in NIS pressure (Fig. 3). No seasonality of detections was apparent (p > 0.2, Table S5).

### Post border

A detailed post-border surveillance strategy based on a statistical risk stratification model ensured detection of NIS was achieved with a minimum power of 0.8 ^13^, the first time this requirement had been enforced by a regulatory authority for detecting terrestrial invasive species. Both Project tenure and undisturbed areas were subject to surveillance for NIS (>140,000 person hours each year). The surveillance system included an estimate of post-border detections by the entire workforce^27^, also a novel component in the QMS. All workers on BWI were trained in biosecurity awareness before going to the island and were actively engaged in biosecurity while on the island. Overall, scientifically-structured biological surveys, which predominantly covered the natural environment adjacent to tenured land, detected 29%, unstructured surveys detected 12% and the contractor workforce, solely on tenured land, made 59% of Project post-border detections. Operationally the detection power ranged 0.8–0.99 ^13^, giving a very high probability of NIS detection.

### Post border detections

Only 141 post-border NIS were detected (Fig. 1D) with about half (48%) associated with movement of people to BWI (Fig. 1E). Ten of the common organisms were commensal invertebrates (e.g. cockroaches) or plant material (Table S6) and were found on Project tenure. A quarter of detections were single organisms (Fig. 2). On average 10,442 inspection hours were required per post-border detection. The frequency distribution observed for border data was similar to that of post-border data (Fig. 2). The log-normal and negative binomial distribution far outweighed the standard Poisson and zero-truncated Poisson distributions for both the positive (non-zero) detections (Table 1) and when the null inspections were included (Table 1). However, the mixture distributions that allowed for both zero-inflation and extreme values provided the best fit (2 extreme values with 100+ organisms; seeds, Fig. 2; AIC_MP_ = 957; AIC_MNB_ = 590; AIC_MLN_ = 424, Table 1).

We found that the mixture log-normal frequency distribution provided the best description of both border and post-border detections. These comprised an additional probability mass for the large number of detections of zero organisms and another for the small number of detections of many organisms, with a possible further probability mass to accommodate single organisms in the post-border detections. Among the non-mixture models, the log-transformed detections were better described by a simple normal distribution and the untransformed data by a negative binomial distribution, than by a standard or zero-inflated Poisson. These distributions should form the basis for sampling programs and for the development of risk assessments at biosecurity borders, in particular the recognition of the large number of zeros, and how that is managed, versus the rare mass incursions. The pattern of non-random and rare mass-contamination involving multiple species has previously been observed for insects and seeds^20, 28, 29^ and can indicate a complex interplay between species-specific entry rates and/or systemic failure in QMS procedures. Incidentally, a long standing hypothesis in invasion ecology^16, 30^ is that there is a 90% reduction in the number of organisms crossing each biosecurity barrier. The ratio of border (1411) to post-border (141) detections corresponds to the so-called 10 s rule observed elsewhere in invasion ecology^3, 30^.

## Discussion

**Figure 4:**
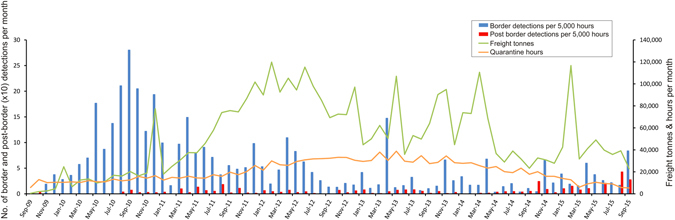
The patterns of quarantine hours, freight tonnage and corresponding border and post-border detections of Non Indigenous Species over the surveillance period. The most compelling correlations are between freight tonnes per month and border detections in the same month (Pearson’s r = 0.65, p < 1E-9), and between freight tonnes per month and quarantine hours in the subsequent month (r = 0.59, p < 1E-5). Correlations that were statistically significant but of smaller magnitude were found between quarantine hours per month and post-border detections in the same month (r = 0.26, p = 0.024) or in the subsequent month (r = 0.27, p = 0.021). Overall, there was an increase of 17.6 quarantine hours (s.e. = 2.5 hours) for every 100 tonne increase in freight in the same month. Post-border detections increased by 0.27 organisms (s.e. = 0.12) for every additional 5,000 quarantine hours in the same month, and by 0.29 organisms (s.e. = 0.12) for every additional 5,000 quarantine hours in the previous month. Monthly rainfall over the period was not strongly or significantly correlated with quarantine hours, border or post-border detections in the same month or subsequent months, with the largest correlation of 0.165 (n.s.) found between rain per month and post-border detections in the subsequent month.

Six years of biosecurity surveillance was sufficient to describe the risk of NIS at the BWI border. While freight volumes varied over the six years, hours of biosecurity activity were maintained while border detections increased rapidly in the first year of QMS operation, and then declined (Fig. 4). The strongest correlation was between freight tonnes per month and border detections in the same month (Pearson’s r = 0.65, p < 1E-9). Imported materials became cleaner as operations and expectations matured, due partly to adaptive learning and auditing processes integral to the QMS. Details of the adaptive learning process and which elements were successful and which were not will be addressed in subsequent publications.

In addition, an increase in post-border detections in 2015 (p < 0.01), may indicate that six years has been sufficient to account for any lag period for the detection of organisms post-border (supported also by Fig. 3, and Table S6). Overall, the commonly detected species post-border were found within three years (Table S6). Post-border detections increased by 0.27 organisms (s.e. = 0.12) for every additional 5,000 quarantine hours in the same month, and by 0.29 organisms (s.e. = 0.12) for every additional 5,000 quarantine hours in the previous month. The presence of a lag period for post-border detections will be tested as surveillance continues in future years. Freight volume (Fig. 4) will decline significantly as construction of the Gorgon Project is completed; however, biosecurity activity will be sustained based on previous learnings.

The types of NIS detected were not always as expected despite five years of planning and assessment of potential invasion pathways, surveillance design and consideration of possible risk species by more than 30 biological specialists and biosecurity experts prior to start of construction in 2009. Firstly, border detections were a poor predictor of post-border detections across all types of organisms except plants. Only three species are present in both Tables S4 and S6. A similar pattern where pre-border detections do not predict establishments has recently been shown for insects^31^. Secondly, some of the more commonly detected post-border NIS (e.g. geckoes) were underestimated, while others considered high risk to islands world-wide (e.g. black rat *Rattus rattus*)^32^ did not eventuate despite high levels observed at ports and supply bases, due to the specific barriers applied (Tables S4 and S6). At the time of writing no establishments of NIS have been detected since the Gorgon Project commenced.

## Conclusions

The example reported here for BWI shows the remarkable scale of effort, and consequently investment, necessary to achieve an effective biosecurity system to protect an area of high conservation value. We also identify the range of elements that should be included in biosecurity systems at national, regional and ecosystem levels, such as sampling based on a three-component mixture log-normal distribution, quantifying the native species that confound border detections, understanding that detections at the border may not predict incursions, and engaging the community and workforce, all individuals working in an area, to improve the level of surveillance. Significant expenditure is being put towards the conservation of biodiversity around the world. However, previous analyses of the efforts made by industries to conserve biodiversity do not include biosecurity^33, 34^. In the BWI QMS described here, a high level of biosecurity planning, implementation and surveillance is justified by exceptional conservation value. Human development has an expanding footprint to more and more remote regions of the world and global change may lead to the opening of previously inaccessible areas such as the Arctic and Antarctic. If hydrocarbon, mineral exploitation, tourism and other trade activities are to be considered for other relatively pristine areas around the world, then it is clear such developments should only proceed with sufficient biosecurity regulation and management to prevent incursions leading to NIS establishment and subsequent loss of native biodiversity.

## Materials and Methods

The location of Barrow Island (BWI) and its infrastructure development, the Gorgon Project, environment, flora and fauna have been described in detail^17^. Gas from the off-shore Gorgon and Jansz–Io fields will be processed in the Gorgon Liquefied Natural Gas (LNG) plant on the 235 km^2^ BWI. Gorgon work sites comprised 1.3% of the land area of BWI.

All access to BWI during construction of the LNG plant was controlled and subjected to biosecurity measures. During construction, commissioning and operation, the Gorgon Project required the transfer of equipment, materials, personnel, marine vessels and aircraft to BWI. The Quarantine Management System^26, 35^ (QMS) was developed to prevent the establishment of non-indigenous terrestrial species and marine pests to BWI or its surrounding waters. The system addresses invasion by invertebrates, vertebrates, plants and marine invasive pests. An *a priori* risk-based approach for micro-organisms did not identify threats to wildlife and plants that were not addressed in the biosecurity management of the supply chain to BWI. The entire QMS and methods are documented^26, 35, 36^. The development of the QMS included 380 supporting documents, 78 specifications and procedures, and 412 activity steps.

Planning the QMS for BWI started in 2002. A risk-based analysis of the 13 material and passenger pathways to BWI^26^ (Table S2) was developed between 2004 and 2009 ^25^, involving 30 independent subject matter and biosecurity specialists. Each pathway was subject to rigorous risk assessment and the application of barriers to minimise the likelihood of invasion. This analysis is the first to present all possible non-natural invasion pathways to a target location for all types of organisms. The hierarchical risk assessment process identified the threats on each pathway and proposed control strategies to reduce risk to levels acceptable to Government and the community^25^. A risk-based option was adopted for micro-organisms and pathogens as it was not feasible to make detections at the border. The QMS was implemented in September 2009 and has been subject to continuous improvement. A summary of the results of the first six years (to September 2015) is presented here.

The LNG facility on BWI required material from 20 countries world-wide. Forty two percent of material was shipped to BWI from Australian ports. QMS requirements were embedded within procurement, contracting and logistics processes, and is the world’s first fully integrated biosecurity system. Domestic and international fabrication and transport sites were required to manage their facilities to be free from discernible evidence of NIS.

Biosecurity control measures, called ‘barriers’, were implemented through innovative design solutions whenever possible, prior to accepting procedural solutions. A large number of unique biosecurity designs were implemented in the planning process and integrated into the supply chain, including the fabrication of specially designed trailers and cargo containers, dedicated airport passenger terminals, catering facilities, decontamination facilities, marine loading facilities and a special Quarantine Clearance Centre on Barrow Island. Material departing for the island was required to be ‘as-new’, mandated in contracts with thorough cleaning and inspection regimes. Fabrication methods and designs were modified to remove potential contamination points, and to facilitate cleaning and inspection before shipping. The risk of contamination was reduced by a wide range of methods including full custody and control of the entire logistics supply chain, mandating timber packaging and dunnage standards, banning all animal and plant importations, maximising pre-preparation of food prior to transport (to exclude seeds, insects, etc.), fumigation, residual insecticide application, application of ingress barriers, wrapping cargo that could not be containerised or crated, and a multi stage inspection process.

All cargo for BWI was cleaned and inspected twice prior to shipment. The full inspection procedure involved up to four tiers at separate handover points. While shortage of qualified inspectors is a major limitation on national biosecurity systems^37^, this was not the case for this project. The inspections were supported by a document and multi-stage tagging system (Material Management Ticket; MMT) that ensured auditable compliance. Multiple inspections were undertaken during transit to the island. Data were collected for all inspections, providing a measurement of missed inspections/re-inspections and corrective actions to address areas needing improvement. Following inspection, materials were considered to be free from discernible evidence of risk material upon leaving the last port for BWI. However, final verification inspections of cargo were undertaken prior to release from ports on BWI. The results of biosecurity detections reported here are very much lower than would be expected for material crossing international and national boundaries due to these extensive pre-border barriers.

The frequency distribution of organisms detected is based on the MMT as a unit of inspection. Most MMT have a value of zero because no NIS were found during the inspection of cargo. The MMT is, however, effectively an underestimate of the number of inspections as it does not include the time for worker participation and surveillance. It also does not include a quantification of the personnel pathway. However, this is not expected to affect the calculation of dispersion given the very large number of zero values of MMT compared to the number of detections. For practical reasons, some of the counts of organisms were estimated by factors of 10 (scaled to 10 s or 100 s or 1000).

The backstop for the pre-border and border biosecurity measures was a surveillance system to detect any introduced flora and fauna early enough to consider an effective response. The surveillance system was designed to achieve a power of detection of at least 0.8, and was implemented with a wide range of detection methods as well as recognising the proven utility of non-scientists (island workers) to detect potential introductions. As a result, the statistical power of detection for the implemented surveillance system approached 0.99 ^27, 38–40^.

Species, including morpho-species were identified by taxonomic specialists at Curtin University or the Department of Agriculture and Food, Western Australia. Over 2,600 invertebrate species were catalogued with photomontage imaging. Examples of invertebrate species can be found at http://www.padil.gov.au/barrow-island/search?queryType=all (accessed 11 April 2016). Vertebrate tissue, scat, hair and saliva analysis for identification was undertaken using molecular methods developed by Helix Pty Ltd.

Data were summarised for border and post-border detections using baselines of total number of MMT. For each of the border and post-border datasets, the observed distribution of counts per detection was compared with zero-truncated Poisson (ZTP), zero-truncated negative binomial (ZTNB) and log-normal (LN) distributions. After including 150,000 border and 10,000 post-border inspections for which no detection was recorded, the distribution of counts per inspection was compared with standard Poisson (P), zero-inflated Poisson (ZIP) and negative binomial (NB) distributions. Since both the border and post-border datasets exhibited not only zero-inflation, but also a small number of extreme values, three-component mixture distributions were also considered, in which the data were represented by a point mass at zero (zero-inflation), an additional point mass for extreme values, and a standard Poisson, negative binomial or log-normal (denoted MP, MNP and MLN, respectively) for the non-zero, less extreme values. A further component to describe the many detections of single organisms was also considered.

The distributions were compared both visually and with respect to Akaike Information Criteria (AIC) and its comparable value for the LN transformed data (AIC + determinant, i.e., AIC−2Σlog *y*). Evidence for one model (model M1, say) compared with another model (model M2) was quantified by exp(AIC_M2_−AIC_M1_)/2)^41, 42^.

## Electronic supplementary material

Supplementary Information
