# Supplementary material for: Zero-tolerance biosecurity protects high-conservation-value island nature reserve
Source: Sci Rep. 2017 Apr 10;7:772. doi: 10.1038/s41598-017-00450-y (PMC5428405; doi:10.1038/s41598-017-00450-y)
Supplement: Supplementary file 1 — Supplementary Information [file 41598_2017_450_MOESM1_ESM.pdf]

Supplementary Materials for

Zero-tolerance biosecurity protects high-conservation-value island nature reserve

John K. Scott, Simon J. McKirdy, Johann van der Merwe, Roy Green, Andrew A. Burbidge, Greg Pickles, Darryl Hardie, Keith Morris, Peter Kendrick, Melissa L. Thomas, Kristin L. Horton, Simon M. O'Connor, Justin Downs, Richard Stoklosa, Russell Lagdon, Barbara Marks, Malcolm Nairn, Kerrie Mengersen

Correspondence to: S.McKirdy@murdoch.edu.au

Table S1. Quantities of material sent to Barrow Island (BWI) between September 2009 and September 2015 for oilfield operations and construction of the LNG plant, and measurement units of the biosecurity system (QMS).

Category of material	Definition	Amount	Units
Twenty foot equivalent units (TEU)	TEU is used to measure a ship's cargo carrying capacity. The dimensions of one TEU are equal to that of a standard 20′ shipping container	205,298	Number
Voyages (domestic)	Voyages of vessels from Australian ports	4,403	Number
Voyages (international)	Voyages of vessels from non-Australian ports	331	Number
Containers	A shipping container is a container with strength suitable to withstand shipment, storage, and handling	63,927	Number
Flat racks	Open-topped, open-sided units that fit into existing below-deck container cell guides and provide a capability	26,048	Number

	for container ships to carry oversized cargo and wheeled and tracked vehicles		
Freight	All materials transported to BWI (excluding rock, sand and aggregate)	8,979,396	Tonnes
Freight (rock, sand, aggregate)	All rock, sand and aggregate transported to BWI	3,182,642	Tonnes
Flights	Aircraft movements from Perth and Karratha to BWI	12,332	Number
Air passengers	Passenger transported by air to BWI	693,781	Number
Material management ticket (MMT)	Document system to track the movement of materials within the supply chain. Each MMT might include one to several containers or similar groupings for inspection purposes	134,265	Number
Biosecurity inspections	Inspection of materials and personnel by biosecurity inspectors	1,472,379	Hours

Table S2. Pathways assessed to manage the likelihood of introductions

Pathway	Description
Personnel and luggage	All air crew, cabin staff, and passengers that travel to BWI and their luggage, tools and toolboxes.
Skid, steel and loose equipment	Items such as; gas turbines, generators, starter/helper motors, compressors, transformers, control panels, electric motors, sheet metal, and fabricated steel components
Modules	Large pre-fabricated LNG plant components constructed in international construction yards and shipped to BWI
Food and perishables	All food and material associated with food consumption sent to BWI
Containerised goods	Items that were inspected and packed into containers/crates for transportation
Sand and aggregate	Comprises road base, backfill, sand and aggregate
Special and sensitive equipment	Items that have been made biosecurity compliant utilising specific barriers. These items cannot be washed, cleaned or chemically treated in a conventional sense, and due to its nature, composition or intended use, requires special handling

Plant and mobile equipment	Includes tracked and wheeled vehicles, earthmoving machinery, welders, compressors, work platforms, cranes, contractor tools and trailers on which equipment may be transported to BWI
Aircraft	Includes both passenger and freight aircraft
Airfreight	All freight transported by aircraft
Helicopter transfers	Transport of personnel from offshore facilities and neighbouring islands to BWI
Domestic vessel	All vessels arriving at BWI and its neighbouring waters from Australian ports
Direct shipment	All vessels arriving at BWI and its neighbouring waters from international ports

Table S3. Number of false positives.

Type of false positive	Number
Species indigenous to BWI	2,143
NIS already established on BWI	405
Non-viable invertebrate NIS*	798
Not applicable (includes procedural deviations, non-viable plant material, soil and organic matter)	201
Vagrant species (i.e. that move naturally between the mainland and the island)	104
Uncertain (biological material that cannot be identified to a species level. Some specimens were too immature or were too damaged for further identification)	776
**Total**	**4,427**

*specimen was dead or only body parts of a specimen were detected.

Table S4. The 20 most abundant species detected at the border and their frequency of detection over the six years.

Species name	Family	Type of organism	2009-2010	2011	2012	2013	2014	2015	Total
Typha sp.	Typhaceae	Plant	161	97	152	69	51	15	545
Cortaderia sp.	Poaceae	Plant	83	28	9	3	2	1	126
Sonchus sp.	Asteraceae	Plant	29	17	12	43	11	1	113
Conyza sp.	Asteraceae	Plant	19	3	7	11	1	1	42
Hypochaeris sp.	Asteraceae	Plant	6	12	8	10	5		41
Senecio sp.	Asteraceae	Plant	2	1	5	7		2	17
Hasarius adansoni	Salticidae	Spider		14	1	1			16
Theba pisana	Helicidae	Mollusc	1	1	6	2	2	1	13
Cenchrus sp.	Poaceae	Plant	1	6	5		1		13
Prietocella barbara	Helicidae	Mollusc		1	5	3	2		11

Hemidactylus frenatus	Gekkonidae	Gecko	1	6	4				11
Saccharum sp.	Poaceae	Plant				3	7	1	11
Lactuca sp.	Asteraceae	Plant			1	8	1		10
Malus domestica	Rosaceae	Plant		4	4	1		1	10
Oryzaephilus sp.	Silvanidae	Beetle		1		3	2	3	9
Pennisetum sp.	Poaceae	Plant	1		4	4			9
Sitophilus oryzae	Curculionidae	Beetle			1	2	1	4	8
Drymaplaneta semivitta	Blattidae	Cockroach		1	1	3	1	1	7
Calomyrmex sp.	Formicidae	Ant				7			7
Tribolium sp.	Tenebrionidae	Beetle	2	2	2			1	7
Total			306	194	227	180	87	32	1026

Cumulative species	11	16	18	20	20	20	20

Notes: *Rattus* sp. Scats were also detected 6 times without any evidence of recent rat presence.

Table S5. Seasonal differences in the number of individuals and type of organism detected presented on a quarterly basis for the period 2010 – 2014.

Year	Type of organism detected	Quarter 1 (Jan. – March)	Quarter 2 (April – June)	Quarter 3 (July – Sept.)	Quarter 4 (Oct. – Dec.)
2010	Seed	9	63	156	176
	Invertebrate	28	47	20	26
	Vertebrate	1	3	2	3
	Plant material*	1	14	11	3
2011	Seed	59	80	53	84
	Invertebrate	82	227	145	315
	Vertebrate	1	6	8	16
	Plant material	2	2	5	11
2012	Seed	29	177	66	35
	Invertebrate	496	472	292	441

	Vertebrate	13	7	12	12
	Plant material	23	12	29	27
2013	Seed	31	108	26	52
	Invertebrate	543	248	180	144
	Vertebrate	26	13	9	17
	Plant material	31	98	44	30
2014	Seed	31	38	27	33
	Invertebrate	282	134	90	88
	Vertebrate	12	10	2	4
	Plant material	32	50	15	14

* excluding seed

Table S6. The 17 most abundant Non-Indigenous Species (occurrence >1) detected post-border and their frequency over the six years.

Species name	Family	Type of organism	2009-2010	2011	2012	2013	2014	2015	Total
Sonchus sp.	Asteraceae	Plant	1	9	4	2		6	22
Ommatoiulus moreletii	Julidae	Millipede	1	2	2	3	3	2	13
Blattella germanica	Blattellidae	Cockroach			3	8			11
Drymaplaneta semivitta	Blattidae	Cockroach		1	1	3	2		7
Medicago polymorpha	Fabaceae	Plant		1		1	2	3	7
Badumna sp.	Desidae	Spider		1	1	2	1	1	6
Arctotheca calendula	Asteraceae	Plant			1	2	1	1	5
Cenchrus sp.	Poaceae	Plant		1	4				5
Cimex lectularius	Cimicidae	Insect				4			4
Conyza sp.	Asteraceae	Plant	1		3				4
Leontodon saxatilis	Asteraceae	Plant		1				2	3

Cornu asperum	Helicidae	Mollusc			1	1	1		3
Litoria rubella	Hylidae	Frog		1	2				3
Supella longipalpa	Blattellidae	Cockroach				1	2		3
Epiphyas postvittana	Tortricidae	Moth		2					2
Lactuca sp.	Asteraceae	Plant						2	2
Christinus marmoratus	Gekkonidae	Gecko	1		1				2
**Total**			4	19	23	27	12	17	102
**Cumulative species**			4	10	14	16	16	17	17
